# Development, implementation, and evaluation process of professional practice models for pediatric nursing: a scoping review protocol

**DOI:** 10.11124/JBIES-25-00378

**Published:** 2026-05-18

**Authors:** Céline Lomme, Gwenaelle De Clifford-Faugère, Cécile Jaques, Anne-Sylvie Ramelet

**Affiliations:** 1 University Institute of Higher Education and Research in Healthcare, Faculty of Biology and Medicine, University of Lausanne, Lausanne, Switzerland; 2 Department Women-Mother-Child, Lausanne University Hospital, Lausanne, Switzerland; 3 Medical Library, Lausanne University Hospital and University of Lausanne, Lausanne, Switzerland; 4 JBI Western Switzerland Best Evidence and Practice Centre, Lausanne, Switzerland

**Keywords:** nursing practice model, pediatric nursing, professional practice model, scoping review

## Abstract

**Objective:**

This scoping review will examine the characteristics of the development, implementation, and/or evaluation of a professional practice model (PPM) for pediatric nursing in Westernized countries.

**Introduction:**

PPMs in nursing are designed to improve excellence in nursing practice and have been shown to improve the work environment. Despite the comprehensive insights from the literature on this topic, it predominantly focuses on adult care settings, leaving a gap in the literature regarding pediatric-specific PPMs.

**Eligibility criteria:**

This review will consider evidence sources that include PPMs developed, implemented, or evaluated in at least 1 inpatient pediatric unit admitting children aged from birth to 18 years in Westernized countries. This review will exclude PPMs established solely in adult care contexts and PPMs developed for specific nursing roles.

**Methods:**

This review will follow the JBI methodology for scoping reviews. Evidence sources using any study design published from 2015 up to the date of the search in any language will be considered. A 3-step search strategy will be used to search CINAHL (EBSCOhost), Embase, PubMed, Web of Science Core Collection, ProQuest ABI/inform collection, Business Source Premier (EBSCOhost), ProQuest Dissertations and Theses, and BASE. Complementary searches will be conducted in Google Scholar, and backward and forward citation searching of included evidence sources will be performed. Two reviewers will independently perform study selection and data extraction, and any disagreements will be resolved with a third reviewer. Data extraction and analysis will be performed using a chart to compare patterns, trends, and gaps in the literature.

**Review registration:**

OSF https://osf.io/g3jxz/overview

## Introduction

Nursing is a challenging, demanding, and fulfilling profession that requires the highest level of expertise, especially when caring for patients in vulnerable situations. There is agreement that ensuring the quality of care in hospital environments becomes more and more challenging as the complexity of patient care increases.^1^ Besides nursing expertise, a dynamic and stimulating work environment is necessary to ensure continuous, high-quality care and patient outcomes. One of the main challenges of nursing worldwide is to strengthen the development of the professional nursing discipline and improve practice.^2^ This is especially true when there are significant changes within hospital organizations. Implementing a professional practice model (PPM) impacts the nursing work environment, job satisfaction, and quality of care.^3^

PPMs for nursing have been defined as frameworks that guide professional behavior, increase control over the delivery of care and the care environment, and enable nurses to provide higher-quality levels of care, which lead to better patient outcomes.^4^ PPMs reflect nursing values and define the structures and processes that enable nurses to manage the care environment and communicate their practice.^5^ A PPM outlines an operational approach and expresses a philosophy of care.^6^ Moreover, it contributes to excellence of care and is one of the requirements to be selected for Magnet designation by the American Nurses Credentialing Center.^7^,^8^

A study by Kirwan *et al*.^9^ noted improvements in the excellence of nursing practice, such as elevated clinical care outcomes and fewer adverse events, after the implementation of a PPM. The literature suggests a positive impact on specific quality indicators, such as pain assessment, pressure ulcers, falls, and hospital-acquired infections; however, further research is required.^10^ Likewise, Zeleníková and colleagues found that a healthy practice environment leads to fewer missed nursing care incidents and better patient outcomes.^11^ In addition, the perception of security and satisfaction of patients and families improves.^9^

The successful implementation of a PPM also impacts the work environment. The literature suggests a positive effect, as stress, burnout, and absenteeism numbers decrease after the implementation.^12^ Work satisfaction, nurse engagement, and retention of new employees also increased after the implementation of this shared conceptual framework.^12^ These findings are supported by Stallings-Welden and Shirey, who described the implementation of a PPM as having a statistically significant impact on registered nurse turnover.^10^

Despite the extensive insights provided in the existing literature on PPMs, most of the research focuses on adult care settings.^4^-^6^ This leaves a notable gap regarding pediatric-specific PPMs.^13^ Cultural differences between pediatric and adult care arise from the unique physiological, developmental, and psychosocial needs of children, who are unable to provide informed consent and require the active involvement of their parents or legal guardians. Health care workers in pediatrics must prioritize advocacy, which involves representing and acting on behalf of children, who are inherently a vulnerable population. Due to these specific needs and the family-centered approach required in pediatric care, pediatric nursing differs significantly from adult nursing and demands a specialized approach.

The existing literature describes the development, implementation, and evaluation of PPMs within adult care, describing an improvement in nursing practice and patient outcomes.^4^-^6^ However, no scoping or systematic reviews have been published on the development, implementation, or evaluation of PPMs specific to pediatric nursing to date. A preliminary search of major registries, including PROSPERO and OSF, was conducted and no current or in-progress scoping reviews or systematic reviews on the topic were identified, indicating a gap in the literature. To address that gap, this scoping review will examine the characteristics of developing, implementing, or evaluating a PPM for pediatric nursing in Westernized countries. These settings have been selected for their comparable health systems, which underpin similar care models and standardized approaches to health care professional training. More specifically, the objectives are to i) identify the characteristics (underlying theories and core components) involved in developing a pediatric nursing PPM in Westernized countries, ii) examine strategies for implementing pediatric PPMs in Westernized countries, and iii) identify evaluation strategies and patient-, family-, and nurse-related outcomes of pediatric PPMs in Westernized countries.

Overall, this review will map and synthesize the available evidence on PPMs for pediatric nursing in Westernized countries to ultimately inform the future development of a PPM for the pediatric hospital setting.

## Review questions

- What are the different steps of a pediatric PPM development process in Westernized countries?
- What are the characteristics (underlying theories and core components) involved in the development of pediatric PPMs in Westernized countries?
- What are the barriers and facilitators of pediatric PPM implementation in Westernized countries?
- What are the pediatric PPM implementation strategies in Westernized countries?
- How is pediatric PPM implementation evaluated, and what types of outcomes (patient-, family-, and nurse-related outcomes) have been reported in the literature when implementing a pediatric nursing PPM in Westernized countries?

## Eligibility criteria

### Participants

This review will consider evidence sources that include licensed nurses and allied health care professionals from any background who work in clinical practice or other roles (eg, management, research, education). In addition, studies including pediatric patients (children from birth to 18 years) and their families, if involved in PPM development, implementation, or evaluation in pediatric hospital settings, will be considered.

### Concept

This review will consider studies that address PPMs, using the commonly accepted operational definition that PPMs provide frameworks that guide professional behavior, enhance control over the delivery of care and the care environment, and enable nurses to deliver higher-quality care, ultimately leading to better patient outcomes.^4^ The scope of this review will encompass all types of information related to the development, implementation, and evaluation of PPMs. It is essential to include these 3 variables—the development, implementation, and evaluation of PPMs—to understand how each component influences the others and the model's effectiveness.

To clarify what constitutes PPMs, we refer to the 7 core components identified and defined by Slatyer and colleagues, which represent the “essential elements of these models.”^5^^(p.144)^ The 7 core components are research/innovation, leadership, independent practice, collaborative practice, environment, development and recognition, and patient outcomes.^5^ Not all core components need to be present in a PPM. The identification of core components will be conducted inductively, using the literature source’s definitions as guiding criteria for determining which PPM elements align with the core components outlined by Slatyer *et al*.^5^

Barriers and facilitators to implementation, as well as implementation strategies, will be defined according to the taxonomy proposed by Powell *et al*.^14^ The outcome measures contain nurse-, patient-, and family-related outcomes. Articles that use a PPM as a framework only to evaluate practice will be excluded. Likewise, PPMs developed specifically to support or implement particular roles (eg, advanced practice nurses, other specialized positions) will be excluded, as their focus does not reflect the broader scope of pediatric nursing practice.

### Context

This scoping review will consider evidence sources from pediatric hospital settings in Westernized countries in which a PPM was developed, implemented, or evaluated. The review will contain information on the type of institution (public, private, mixed, non-governmental, religious) if this detail is reported in the included evidence sources. This review will exclude PPMs established in lower- and middle-income countries, as defined by the World Bank classification, or PPMs established solely in adult care contexts.^15^

### Types of sources

This review will consider all study designs as well as gray literature. Published reviews, journal articles, conference presentations, guidelines, expert consensus papers, and policy documents will be considered. Editorials, letters, and conference abstracts without conference proceedings will be excluded.

## Methods

The proposed scoping review will be conducted according to the JBI methodology for scoping reviews^16^ and reported using the Preferred Reporting Items for Systematic Reviews and Meta-Analyses extension for Scoping Reviews (PRISMA-ScR).^17^ This protocol has been registered in OSF (https://osf.io/g3jxz/overview).

### Search strategy

The search strategy will aim to locate both published and unpublished evidence sources and will be developed in collaboration with a librarian (CJ). A 3-step search strategy will be conducted. The first step was an exploratory search in PubMed and CINAHL (EBSCOhost). This was followed by an analysis of the keywords contained in the titles and abstracts, as well as the indexing terms used to describe the relevant articles found. The second step consists of developing search strategies using identified vocabulary for the following databases: PubMed, Embase (Embase.com), CINAHL (EBSCOhost), Web of Science Core Collection, ProQuest ABI/inform collection, Business Source Premier (EBSCOhost), ProQuest Dissertations and Theses (ProQuest), and BASE (Bielefeld Academic Search Engine). Sources of gray literature to be searched include the websites of renowned children’s teaching hospitals from North America, Europe, and Australasia. We will also consult official organizational websites and membership directories, as well as recognized ranking platforms, which will provide an authoritative list of affiliated institutions. See Appendix I for the list of hospitals that will be contacted. Complementary searches will be carried out in Google Scholar, with the first 200 results screened within the search engine itself. All search strategies will be peer-reviewed by another librarian using the Peer Review of Electronic Search Strategies (PRESS) checklist.^18^ The third step will incorporate backward and forward citation searching of included evidence sources and relevant reviews. A sample search strategy for CINAHL (EBSCOhost) is presented in Appendix II.

No language limits will be applied to the search strategy. Given the multilingual competencies of the review team members, evidence sources published in languages other than English will be considered for inclusion where feasible. Sources published in languages outside the review team’s language proficiency will be excluded from the review. No professional translation services will be employed, and the exclusion of non-accessible languages will be acknowledged as a methodological limitation. The search strategy will be restricted to the past 10 years (2015 to the present) to ensure that the development and implementation strategies reflect a contemporary health care context. This time frame will also ensure that PPMs are aligned with the new vision statement of the Magnet Recognition Program, formulated in 2020, which expanded its focus to a global perspective, emphasizing innovation, interprofessional collaboration, and worldwide impact on care delivery.^8^

### Source of evidence selection

Following the search, all identified citations will be collated and uploaded into EndNote v.20.4.1 (Clarivate Analytics, PA, USA) and duplicates removed. The 2-phase screening process will be piloted on 50 records by 2 reviewers independently to ensure their consistency in screening. Following the pilot test, titles and abstracts will be screened by the 2 reviewers independently for assessment against the eligibility criteria for the review. Potentially relevant papers will be retrieved in full and their citation details imported into Rayyan (Rayyan Systems Inc., Cambridge, MA, USA). The full text of selected citations will be assessed in detail against the eligibility criteria by 2 reviewers independently. Reasons for exclusion of full-text papers that do not meet the eligibility criteria will be recorded and reported in the scoping review. Any disagreements that arise between the reviewers at each stage of the selection process will be resolved with a third reviewer. Authors of evidence sources will be contacted once to request missing or additional data, where required. In the absence of a response, a follow-up contact attempt will be made. If it goes unanswered, the source will be included in the analysis with a note indicating the lack of response as a limitation. The results of the search will be reported in full in the final scoping review and presented in a PRISMA flow diagram.^19^

### Data extraction

Data will be extracted from evidence sources included in the scoping review by 2 reviewers independently using a data extraction tool developed by the reviewers. Any disagreements that arise between the reviewers will be resolved with a third reviewer. The data extracted will include specific details about the article characteristics (authors, year of publication, country of origin, type of article, title, aim, study design, and setting); PPM development characteristics (underlying theories and core elements); PPM implementation (strategies, barriers, and facilitators); evaluation of PPM implementation; and evaluation of patient-, family-, and nurse-related outcomes relevant to the review questions. A draft extraction tool is provided (see Appendix III).^17^ This data extraction tool will be pilot tested on a minimum of 3 sources, or more if modifications are needed. If modifications are deemed necessary by the reviewers during the data extraction process, revisions will be clearly detailed in the final scoping review.

### Data analysis and presentation

Data analysis will be performed to summarize patterns, trends, and gaps in the literature. The results will be presented in narrative and tabular formats, as well as maps and diagrams, where appropriate, to visually represent the data. The development process will be described using a descriptive tabular format, with a narrative summary explaining commonalities and variations across evidence sources. Theoretical frameworks underpinning the PPMs will be synthesized and presented through a structured narrative descriptive table summarizing key theoretical characteristics. Core components will be presented in a separate descriptive table. Implementation strategies, including barriers and facilitators, will be narratively presented in a tabular format, and trends and gaps will be analyzed and visually represented. Outcome measures will be mapped with a diagram, narratively described, and analyzed for patterns.

## Acknowledgments

This scoping review protocol contributes to a PhD in nursing science degree at the University of Lausanne, Switzerland for CL.

GDF holds a Canadian Institutes of Health Research (CIHR) postdoctoral scholarship.

## Funding

The Lausanne University Hospital and the University of Lausanne partially support this project through a doctoral fellowship and salary of CL. The funders had no role in the design, conduct, analysis, or reporting of this protocol or the resulting scoping review.

## Global priority and equity alignment

This scoping review aligns with the United Nations Sustainable Development Goal (SDG) 3, specifically target 3.c, which aims to strengthen health financing and the recruitment, development, training, and retention of the health workforce. By synthesizing evidence on complex interventions and their implementation and evaluation strategies, this review contributes to informing workforce development and capacity-building approaches that support high-quality, sustainable health systems.

The review also aligns with SDG 9, particularly target 9.1, by examining interventions that contribute to the development of resilient, innovative, and inclusive health system infrastructures. By identifying core components, implementation strategies, and outcome measures, the review supports knowledge generation to enhance equitable access to effective and sustainable health services, thereby promoting health system resilience and innovation across diverse contexts.

## Author contribution

CL: conceptualization, methodology, writing (original draft). GDC-F: conceptualization, methodology, writing (review and editing). CJ: methodology, writing (review and editing). A-SR: conceptualization, methodology, writing (review and editing), supervision. All authors have read and approved the final submitted version of the protocol.

## Appendix I: List of hospitals to be contacted

### Europe

**Table:**

Hospital	Location
Erasmus MC-Sophia Children’s Hospital	Rotterdam, Netherlands
Great Ormond Street Hospital for Children	London, United Kingdom
HUS New Children's Hospital	Helsinki, Finland
Necker-Enfants Malades Universitary Hospital AP-HP	Paris, France
Oslo University Hospital	Oslo, Norway
Sant Joan de Déu Barcelona Children’s Hospital	Barcelona, Spain

European Children’s Hospitals Organisation. Members [internet]. Esplugues de Llobregat: ECHO; n.d. [cited 2025 Oct 21]. Available from: https://www.echohospitals.org/members.

### North America

**Table:**

Hospital	Location
Alberta Children’s Hospital Foundation	Calgary, AB, Canada
Boston Children's Hospital in Boston, MA	Boston, MA, USA
Children's Hospital Colorado in Aurora, CO	Aurora, CO, USA
Children’s Hospital of Philadelphia CHOP	Philadelphia, PA, USA
Children's National Hospital in Washington, DC	Washington, DC, USA
CHU Sainte-Justine Foundation’s	Montréal, QC, Canada
Hospital for Sick Children Toronto	Toronto, ON, Canada
Montreal Children’s Hospital	Montréal, QC, Canada
Seattle Children's Hospital in Seattle, WA	Seattle, WA, USA

Canada’s Children’s Hospital Foundations. Member foundations [internet]. CCHF; n.d. [cited 2025 Oct 21]. Available from: https://childrenshospitals.ca/member-foundations/. U.S. News and World Report. Best children's hospitals: national rankings [internet]. U.S. News and World Report; n.d. [cited 2025 Oct 21]. Available from: https://health.usnews.com/best-hospitals/pediatric-rankings.

### Australasia

**Table:**

Hospital	Location
Children's Health Queensland Hospital	Brisbane, QLD, Australia
Perth Children's Hospital	Perth, WA, Australia
Royal Children's Hospital Melbourne	Melbourne, Vic, Australia
Starship Children’s Hospital	Auckland, New Zealand
Sydney Children's Hospitals Network	Sydney, NSW, Australia

Children’s Healthcare Australasia. Home [internet]. CHA; n.d. [cited 2025 Oct 21]. Available from: https://children.wcha.asn.au/.

## Appendix II: Search strategy

The initial search was conducted on October 22, 2024, in CINAHL (EBSCOhost) with 105 records retrieved.

The search strategy uses 2 approaches combined with OR

Approach 1: The first line searches for the concept “professional practice model.” This line is then combined with “AND” to the second line searching the “pediatric context.”

Approach 2: The third line searches for the expression “professional practice model” in the title only to retrieve important references on the subject which may discuss the pediatric context only in the full text.

((((MH “Professional Practice” OR MH “Nursing Practice+”) AND MH “Nursing Models, Theoretical+”) OR ((TI “professional practice model*” OR AB “professional practice model*”) OR (TI “nursing excellence”) AND (TI nurs* OR AB nurs* OR SU nurs*)) OR TI “nursing practice model*” OR AB “nursing practice model*”)

**AND** (MH “Hospitals, Pediatric” OR MH Pediatrics+ OR MH “Pediatric Nursing+” OR MH Child+ OR TI paediatric* OR AB paediatric* OR TI pediatric* OR AB pediatric* OR TI child OR AB child OR TI children* OR AB children* OR TI infan* OR AB infan*))

**OR** (TI “professional practice model*” AND nurs*)

## Appendix III: Draft data extraction instrument

**Table:**

Study characteristics	Extracted data
Author(s)	
Year of publication	
Country of origin	
Type of article/study design	
Title	
Aim and objective	
Context	
Definition of PPM	
Core elements	
Setting	
Development of PPM (theory used and development process)	
Implementation (strategies, barriers, facilitators)	
Evaluation of PPM implementation	
Process outcomes	
Nursing-sensitive outcomes	
Patient and family outcomes	

PPM, professional practice model.
